# Common Diagnoses from Surgical Biopsies and Investigation of Leporipoxvirus in Pet Rabbits (*Oryctolagus cuniculi*) in Taiwan

**DOI:** 10.3390/ani15091234

**Published:** 2025-04-27

**Authors:** Ya-Mei Chen, Yang-Chun Wu, Ching-Liang Kuo, Wei-Hao Lin, Kuo-Ping Shen

**Affiliations:** College of Veterinary Medicine, National Pingtung University of Science and Technology, Neipu 912301, Taiwanwhlin@mail.npust.edu.tw (W.-H.L.); shenkp@mail.npust.edu.tw (K.-P.S.)

**Keywords:** fibroma, fibrosarcoma, *leporipoxvirus*, rabbits, tumor

## Abstract

This study investigated common diagnoses from surgical biopsies of domestic rabbits (*Oryctolagus cuniculi*) in Taiwan and examined the role of leporipoxvirus in tumor pathogenesis. Biopsy specimens from 70 rabbits collected between 2014 and 2023 were retrospectively analyzed, yielding 85 diagnoses. Polymerase chain reaction (PCR) was performed to detect leporipoxvirus in formalin-fixed, paraffin-embedded tissues diagnosed with fibroma, fibrosarcoma, or myxosarcoma. The most commonly affected systems were the integumentary (*n* = 41) and reproductive (*n* = 36) systems. Common integumentary tumors included fibrosarcomas (*n* = 12), trichoblastomas (*n* = 8), mammary gland tumors (*n* = 5), and fibromas (*n* = 4). In the reproductive system, the most common lesions were uterine adenocarcinomas (*n* = 16), uterine endometrial cystic hyperplasia and hypertrophy (*n* = 5), and uterine adenomyosis (*n* = 4). The 15 cases of fibroma, fibrosarcoma, and myxosarcoma were tested for leporipoxvirus using PCR. No viral sequences were detected in these tumors. This study identified the common diagnoses from rabbit biopsy specimens and found no leporipoxvirus infection in samples of fibrosarcoma and fibroma. This is the first study on tumors in pet rabbit biopsies and the first in Taiwan to investigate leporipoxvirus infection, providing valuable insights for future diagnosis and management.

## Simple Summary

Rabbits are becoming increasingly popular pets in Taiwan, and veterinarians often need to diagnose and treat various health conditions in rabbits. This study analyzed biopsy samples from 70 pet rabbits to identify common diseases and investigate whether a specific virus, leporipoxvirus, plays a role in tumor development. The most frequently affected body systems were the skin and the reproductive system. Skin tumors, such as fibrosarcoma and trichoblastoma, were common in male rabbits, while uterine tumors, especially uterine adenocarcinoma, were more frequent in females. Using a specialized laboratory test (PCR), we examined 15 tumor samples for leporipoxvirus, which has been linked to certain rabbit skin diseases in other countries. No virus was detected in any of the samples. These findings suggest that this virus does not contribute to tumor formation in pet rabbits in Taiwan. Understanding the most common diseases in pet rabbits helps veterinarians improve early diagnosis, provide better treatment options, and guide pet owners in preventive care, such as spaying female rabbits to reduce the risk of reproductive tumors. This study provides valuable insights for rabbit health management and veterinary practice.

## 1. Introduction

European rabbits (*Oryctolagus cuniculi*) are becoming increasingly popular pets in Taiwan. While they have a typical lifespan of 5–10 years [1], this is increasing due to improved knowledge among owners about rabbit care. Consequently, surgical biopsies in pet rabbits have become more common. Currently, no studies have been conducted in Taiwan on the diagnoses obtained from surgical biopsies of pet rabbits. Identifying the most common diagnoses is essential to ensure that veterinarians have sufficient knowledge of the conditions they encounter and can provide the most appropriate treatment.

The leporipoxvirus genus belongs to the *Chordopoxvirinae* subfamily of the *Poxviridae* family. Two viruses in this genus infect rabbits: the Shope fibroma virus and the myxoma virus [2,3]. The Shope fibroma virus causes infectious cutaneous fibromas in both American cottontail rabbits (*Sylvilagus* spp.) and European rabbits (*Oryctolagus cuniculus*) [4]. The myxoma virus originated in South American rabbits and hares, in which it has no serious effects. However, in European rabbits, it induces myxomatosis, a severe and usually fatal disease characterized by cutaneous myxomas [5]. Both viruses are primarily transmitted by arthropod vectors [6]. The vectors of the Shope fibroma virus reported in the United States include mosquitoes (*Aedes aegypti*, *Aedes triseriatus*, *Culex pipiens*, *Culex quinquefasciatus*, and *Anopheles quadrimaculatus*), reduviid bugs (*Triatoma infestans*, *Triatoma phyllosoma,* and *Rhodnius prolixus*), and bed bugs (*Cimex lectularius*) [7]. The incidence is highest between July and November when insect activity is at its peak [1]. Although Shope fibroma is typically reported in North America, a 2024 study was the first to document Shope fibroma in pet rabbits in Mexico [8]. This indicates that the Shope fibroma has spread to other regions. Located in the subtropical region, Taiwan has a high prevalence of mosquitoes and other insects. For instance, *Aedes aegypti*, a common mosquito species, is the primary vector for dengue fever in humans in Taiwan [9]. The local presence of such vectors, combined with the increasing international movement of animals and climate conditions conducive to vector-borne disease, raises concern about the potential introduction and establishment of *leporipoxviruses* in Taiwanese rabbit populations. In Asia, studies from Japan and Hong Kong have reported common cutaneous neoplasms but have not documented any virus-associated lesions. This lack of investigation presents a critical knowledge gap that may hinder the early detection of emerging infectious diseases in exotic pets. The two aims of this study were to characterize and document the histopathological diagnoses obtained from surgical biopsies of pet rabbits in Taiwan and to investigate the potential presence of leporipoxvirus infection in selected mesenchymal tumors, including fibroma, fibrosarcoma, and myxosarcoma.

## 2. Materials and Methods

### 2.1. Case Collection

All cases in this study were biopsy specimens from animal hospitals submitted to the Animal Disease Diagnostic Center (ADCC) at the College of Veterinary Medicine, National Pingtung University of Science and Technology, Taiwan. The specimens were obtained between 2014 and 2023, and the data were retrospectively collected for this analysis. The collected data included species, age, sex, neuter status, lesion location, and lesion size. Information regarding rabbit breed was not provided by the submitting veterinary clinics at the time of biopsy submission and was therefore unavailable for analysis.

### 2.2. Histology

Biopsied tissue was fixed in 10% neutral buffered formalin for no more than 48 h. The fixed tissue was routinely processed, embedded in paraffin, sectioned at a thickness of 4 μm, and then stained with hematoxylin and eosin (HE). All diagnoses were confirmed by two veterinary pathologists based on standard diagnostic criteria described in the relevant peer-reviewed literature.

### 2.3. Polymerase Chain Reaction Tests

Of the 17 fibrocyte-associated tumors, 15 were screened for *leporipoxvirus*. The tumors included fibrosarcomas (*n* = 11), fibromas (*n* = 3), and myxosarcoma (*n* = 1). From each tumor, eight serial 10 μm-thick formalin-fixed, paraffin-embedded (FFPE) tissue sections were collected. The DNA was then purified using a QIAamp^®^ DNA FFPE Tissue Kit (cat. 56404; Qiagen, Germantown, MD, USA) according to the manufacturer’s instructions.

The primers used for polymerase chain reaction (PCR) detection of leporipoxvirus were based on those used in previous studies [3,10]. These were designed for the detection of those members of the *Chordopoxvirinae* subfamily that infect vertebrates, with leporipoxvirus being one of the classified genera. The selected forward primer targets the insulin metalloproteinase-like protein gene, and the reverse primer targets the intracellular mature virion membrane protein gene. The primer sequences used for PCR detection of leporipoxvirus in pet rabbits were adapted from a previous study [1] and are shown in Table 1.

The PCR reaction mixture consisted of 10 μL of Phire Green Hot Start II PCR Master Mix (Thermo Scientific^TM^, Waltham, MA, USA), 1 μL of forward primer, 1 μL of reverse primer, and ultrapure distilled water (DNase- and RNase-free). Sample tubes containing 2 μL each of DNA extracted from the tumor specimens were prepared. The negative controls included only the reaction mixture, while the positive control contained 1 μL of the synthesized genomic DNA. The sequence of the synthesized genomic DNA was gcaccaaaaactcatataacttctagccgtggaggcatttgcgatgaaccgggacacatcgaacgagataagtatatgttccaacagatgtgccgtacccagtatacctcctatgtccttttcgaatccgaacccagaaatccctacgtatatatctttgttcgtggaagtgtttataaatacacgcaccccgttatcgaatacaatcatttactaaggagtaaaatagg.

The following protocol was used for the PCR: an initial cycle at 92 °C for 2 min, 10 cycles at denaturation (92 °C for 10 s), annealing at 50 °C for 30 s, and elongation at 68 °C for 1 min. This was followed by 20 further cycles of denaturation, annealing, and elongation, with each successive elongation step extended by an additional 2 s. PCR amplicons and 100 bp + 3K DNA Ladder DNA marker (Excel-Band™, Smobio Technology, New Taipei City, Taiwan) were then electrophoresed in a 2% agarose gel in 1× tris-acetate-EDTA (TAE) buffer at 100 V for 32 min. The gels were subsequently stained with Healthview™ Nucleic Acid Stain (Genomics BioSci & Tech Co., Ltd., New Taipei City, Taiwan) and imaged on a gel documentation system using MultiAnalyst software version 1.1 (Bio-Rad, Hercules, CA, USA).

## 3. Results

Between 2014 and 2023, the ADCC received 85 biopsy specimens from 70 pet rabbits (Table S1). The median (range) age of the rabbits was 83 (12–149) months. There were 20 adult (6–60 months old) and 50 elderly (>60 months old) rabbits. No biopsy specimens were obtained from young rabbits (<6 months old). Within this sample, 52.9% (37/70) were female and 47.1% (33/70) were male. Among the females, 78% (29/37) were intact, while 21% (8/37) were neutered. Among the males, 75% (25/33) were intact, 18% (6/33) were neutered, and two had unknown neuter status (Table 2).

**Figure 1:**
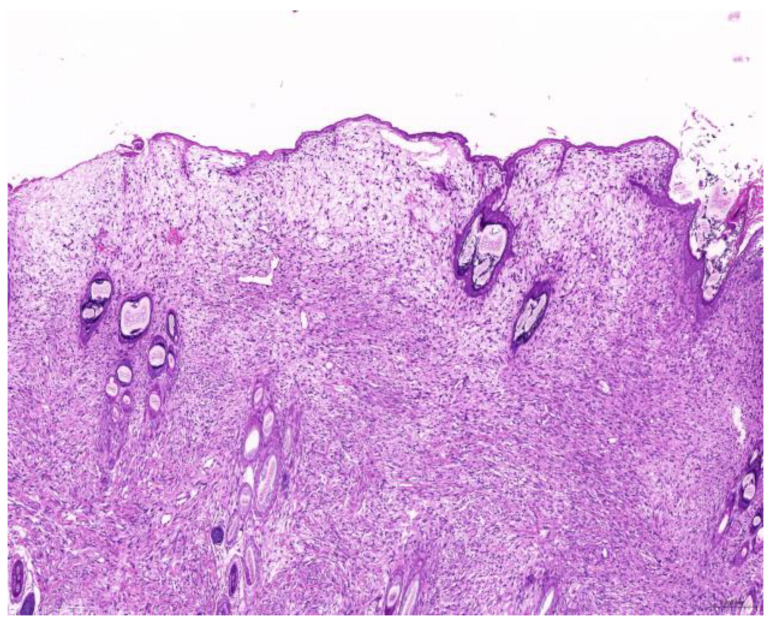
Fibrosarcoma. The neoplasm is located in the dermis and is arranged in streams (scale bar = 200 µm). Hematoxylin and eosin staining.

**Figure 2:**
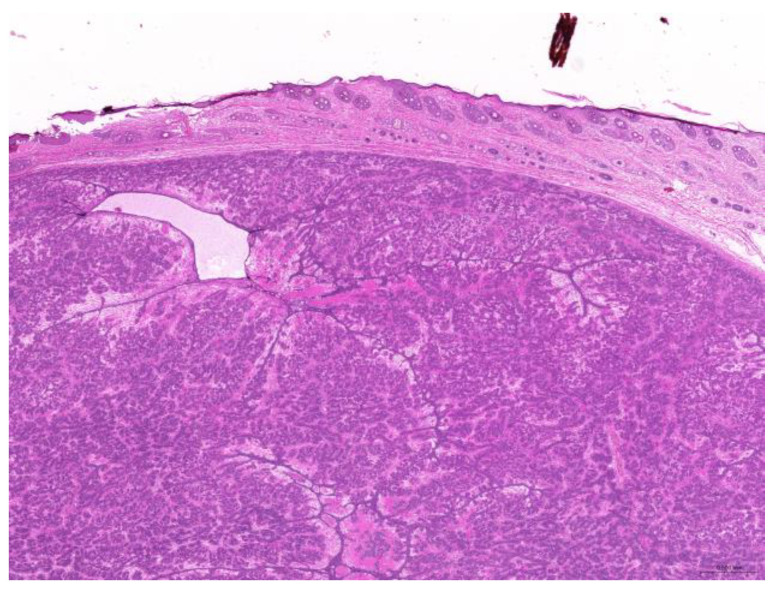
Trichoblastoma. Neoplastic cells are arranged in trabeculae (scale bar = 500 µm). Hematoxylin and eosin staining.

**Figure 3:**
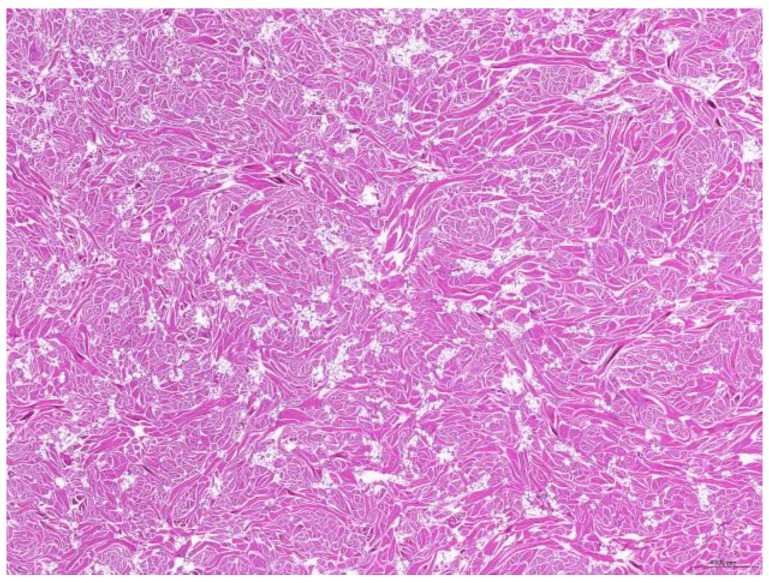
Fibroma. The neoplasm consists of abundant mature collagen fibers (scale bar = 500 µm). Hematoxylin and eosin staining.

**Figure 4:**
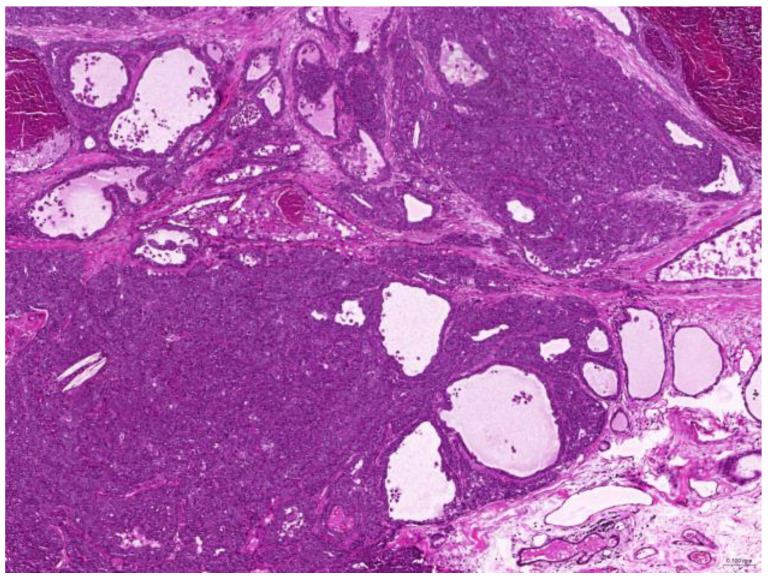
Mammary gland adenocarcinoma. The neoplasm reveals invasive behavior (scale bar = 100 µm). Hematoxylin and eosin staining.

**Figure 5:**
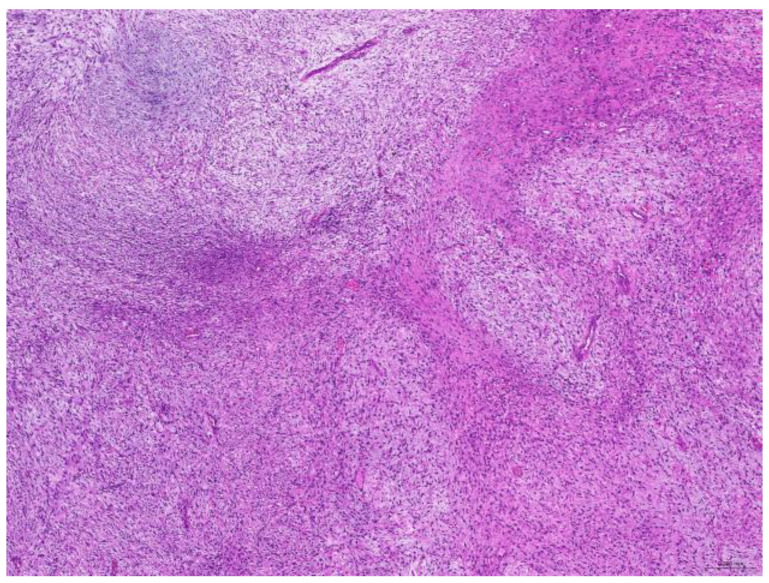
Myxosarcoma. The neoplasm contains basophilic mucinous material (scale bar = 200 µm). Hematoxylin and eosin staining.

Of the 85 biopsy samples, the most commonly affected system was the integumentary system (48.2%, 41/85), followed by the reproductive system (42.4%, 36/85), the digestive system (8.2%, 7/85), and the urinary system (1.2%, 1/85) (Table 3). Of the 41 rabbits with an affected integumentary system, 39 had neoplastic and two had non-neoplastic disorders. The median age of these affected rabbits was 87 (12–149) months. There were 15 females and 26 males, with a female-to-male ratio of 0.54:1. The most common integumentary diagnosis was fibrosarcoma (29.3%, 12/41) (Figure 1), followed by trichoblastoma (19.5%, 8/41) (Figure 2), fibroma (9.8%, 4/41) (Figure 3), lipoma (7.3%, 3/41), mammary gland adenoma (7.3%, 3/41), and mammary gland adenocarcinoma (4.9%, 2/41) (Figure 4), among others. The median age of the rabbits with fibrosarcomas was 108 (54–126) months. Of the 12 cases with fibrosarcoma, 4 were females and 8 were males, with a female-to-male ratio of 0.5:1. The tumors were located on the neck, chest, abdomen, and extremities. The median age of the rabbits with fibromas was 101 (48–108) months. All four of these were males, with tumors exclusively located on the chest. Myxosarcoma was diagnosed in a 108-month-old male rabbit, with the tumor located on the left elbow (Figure 5).

**Figure 6:**
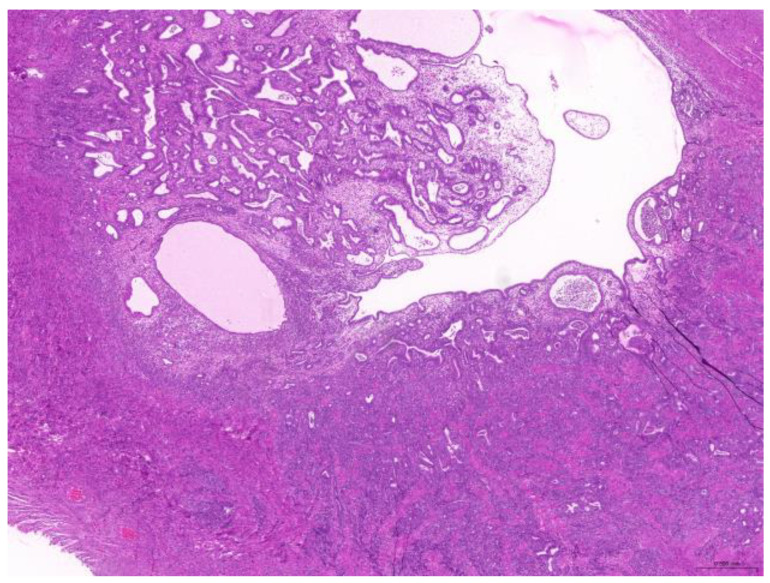
Uterine adenocarcinoma. The neoplasm effaces the original uterine structure (scale bar = 500 µm). Hematoxylin and eosin staining.

Thirty-six biopsy specimens were taken from the reproductive systems of 22 females and two males (a female-to-male ratio of 11:1). All of the females were intact, and 32 diagnoses were obtained from these 22 females. The median age of the affected females was 71 (20–132) months. The most common diagnoses were uterine adenocarcinoma (50%, 16/32) (Figure 6), followed by endometrial cystic hyperplasia (15.6%, 5/32), uterine adenomyosis (12.5%, 4/32), uterine leiomyoma (6.25%, 2/32), and luteoma (6.25%, 2/32). The median age of the females with uterine adenocarcinoma was 82 (42–132) months. From the two male rabbits, four diagnoses affecting the reproductive system were obtained. Both of these males were intact. The median age of the affected males was 113.5 (109–118) months. The most common diagnosis was interstitial cell tumor (50%, 2/4), followed by seminoma (25%, 1/4) and testicular atrophy (25%, 1/4).

The seven digestive system diagnoses were for six animals and comprised six non-neoplastic disorders and one neoplastic disorder. The median age of these six animals was 73 (22–96) months. There were one female and six males, resulting in a female-to-male ratio of 0.17:1. The non-neoplastic disorders included appendicitis, rectitis, anusitis, fatty liver, hepatic hemorrhage and necrosis, bile stasis, and mineral deposition. There was one case of rectal adenocarcinoma. The one case with a condition affecting the urinary system was a neutered male aged 126 months with urothelial carcinoma.

**Figure 7:**
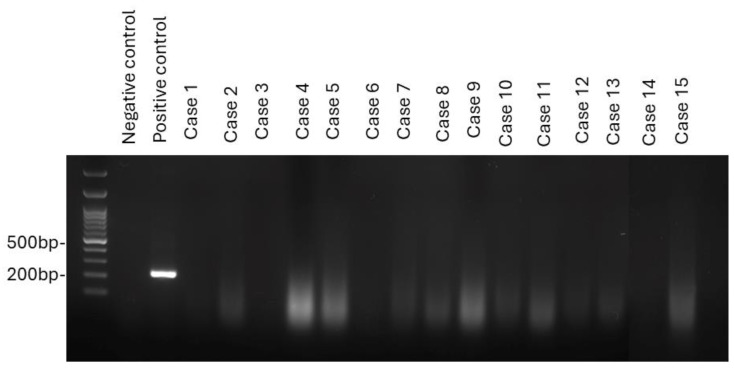
PCR analysis was performed on 15 cases of fibrosarcoma, fibroma, and myxosarcoma to detect *leporipoxvirus*. All cases tested negative for leporipoxvirus DNA.

To understand the role of leporipoxvirus in tumorigenesis, specimens from 15 cases, including 11 with fibrosarcoma, three with fibroma, and one with myxosarcoma, were subjected to virus detection by PCR. Leporipoxvirus DNA was not amplified from any of the 15 tested samples (Figure 7).

## 4. Discussion

This study collected data on 85 diagnoses from 70 pet rabbits, consisting of 37 females and 33 males. The most commonly affected systems were the integumentary and reproductive systems. This finding is consistent with those of previous studies [11,12]. With the exception of lymphoma, most tumors occur in older rabbits [13]. In this study, the median age of the cases was 83 months, and there were no rabbits younger than 6 months. Multiple studies indicate that sex is a risk factor for tumors in pet rabbits [13,14,15]. Female rabbits are more prone to reproductive disorders, whereas males are more likely than females to develop integumentary tumors [13,14,15]. In this study, the female-to-male ratio for integumentary tumors was 0.54:1, while that for reproductive disorders was 11:1. Our results similarly indicated that males were more prone to skin tumors, while females were more likely to have reproductive disorders. In our study, males appeared more prone to integumentary tumors, and this may reflect biological predispositions, such as hormonal influences or behavior. Alternatively, it may be artefactual, since female rabbits are more likely to die from or be euthanized due to uterine adenocarcinomas before developing skin tumors.

Among the integumentary diagnoses, the most common skin tumors were fibrosarcomas, trichoblastomas, mammary gland tumors, and lipomas in our study. Previous studies on tumor prevalence can be categorized by regions. In the United States, a study of 1238 rabbits with neoplastic and non-neoplastic lesions identified trichoblastoma as the most common type of skin tumor (13.1%), followed by fibrosarcoma (4%), and fibroma/collagenous hamartoma (3.3%) [16]. Another study, also conducted in the US, of 672 rabbits with cutaneous neoplasms, found trichoblastoma (20%), unclassified mesenchymal tumors (9.5%), collagen hamartomas (8.4%), squamous papilloma (6.4%), and mammary carcinoma (6%) to be the most common tumors [17]. A third retrospective study of 190 cutaneous neoplasms in pet rabbits in the US, excluding mammary tumors, similarly found trichoblastoma (31.1%) to be the most common, followed by collagenous hamartoma (14.2%), Shope fibroma (10%), lipoma (5.3%), and myxosarcoma (4.7%) [1]. The endemic presence of the Shope fibroma virus in North America likely contributes to the occurrence of virus-associated tumors such as Shope fibroma. These cases provide a contrast to reports from other regions where such viruses have not been confirmed. In Europe, a study conducted in Poland identified trichoblastoma, fibrosarcoma, lipoma, and fibroma as the most common skin tumors [14]. In Europe, the only virus-induced tumors are myxoma and myxosarcoma, both caused by the myxoma virus [6,18]. In Asia, a survey in Japan of 360 rabbits with cutaneous neoplasms found the most common to be cutaneous soft tissue sarcoma (15.1%), mammary gland adenocarcinoma (8.2%), trichoblastoma (4.2%), and mammary gland adenoma (4%) [12]. The soft tissue sarcoma category included undifferentiated sarcoma, fibrosarcoma, peripheral nerve sheath tumor, myxosarcoma, and liposarcoma. Similarly, we found fibrosarcoma, trichoblastoma, and mammary gland tumors to be the most prevalent. This was in concordance with the findings from both Poland and Japan. Recently, a retrospective study in Hong Kong of tumors and tumor-like lesions in rabbits revealed adenocarcinoma (26.4%), trichoblastoma (21.4%), sarcoma (9.4%), and thymoma (8.2%) to be the most common diagnoses from surgical biopsies [19]. To date, no virus-associated tumors have been reported in Asia [18]. In addition to regional differences, there appears to be a sex-related pattern in the occurrence of skin tumors in pet rabbits. Among skin tumors, males seem more prone to developing collagenous hamartomas and cutaneous soft tissue sarcomas [1,12,17]. In our study, the female-to-male ratio for fibrosarcoma was 0.5:1, supporting this observation.

In pet rabbits, Shope fibroma and Shope papilloma are common virus-induced tumors [1]. In our study, fibroma was identified in 9.8% of cutaneous tumors—a relatively high proportion given that no virus-associated tumors have been previously reported in Asia. The Shope fibroma virus typically infects young rabbits but can also affect adults [4]. The median age of rabbits diagnosed with fibroma in our study was 89 (48–108) months. Notably, one fibroma case exhibited a suspected intracytoplasmic inclusion body in an epithelial cell, raising the possibility of viral involvement. Given the presence of known arthropod vectors for Shope fibroma virus in Taiwan, this observation warrants further investigation into whether *leporipoxviruses*, such as the Shope fibroma virus, might be circulating undetected in the local rabbit population. Additionally, our identification of a single case of myxosarcoma, another tumor potentially associated with *leporipoxvirus*, further supports the need to examine a broader range of mesenchymal tumors—specifically fibroma, fibrosarcoma, and myxosarcoma—for evidence of viral infection.

In light of these findings and the lack of previous studies investigating virus-associated skin tumors in pet rabbits in Taiwan, we sought to evaluate the potential involvement of leporipoxvirus through molecular detection techniques applied to archived FFPE tissues. However, leporipoxvirus DNA was not amplified from any of the 15 samples. This result suggests either that *leporipoxviruses* are not present in these tumors or that limitations such as low viral load, DNA degradation, or viral sequence variability may have hindered detection. While the results do not support the current presence of leporipoxvirus in Taiwan’s pet rabbits, the relatively high fibroma incidence and the presence of vectors warrant continued surveillance. The cottontail rabbit papillomavirus can lead to Shope papilloma and squamous cell carcinoma (SCC) in both *Oryctolagus* and *Sylvilagus* species [6]. In this study, only one case of SCC was reported in a 133-month-old male rabbit, with the tumor located on the pinna. Spontaneous SCC in rabbits commonly occurs on the ears and feet [1,20]. McLaughlin reported a case in the United States in which papillomavirus was detected by PCR amplification of viral DNA [20].

In pet rabbits, mammary gland tumors are common in females over 2 years old [13]. These are most often adenocarcinoma, which is likely associated with uterine hyperplasia and hyperestrogenism [11,13,21]. Of the organ systems, the female reproductive tract, particularly the uterus, is the most commonly affected [11,12]. Spaying female rabbits before they reach 6 months is advised to prevent uterine and mammary tumors [13]. In our study, 32 of the diagnoses, obtained from 22 intact animals, affected the female reproductive tract. This supports the aforementioned recommendation, further indicating that neutering is essential for prevention. Common neoplastic and non-neoplastic lesions in the uterus include uterine adenocarcinoma, endometrial hyperplasia, carcinosarcoma, and adenomyosis [12]. In our study, the most common diagnoses were uterine adenocarcinoma (50%), followed by endometrial cystic hyperplasia (15.6%) and uterine adenomyosis (12.5%). Uterine adenocarcinoma has previously been shown to affect 50–80% of female rabbits older than 3 years [13]. The median age of affected females with uterine adenocarcinoma in our study was 82 (42–132) months (6.8 years). It is likely that the prevalence of uterine adenocarcinoma is underestimated in our study, as these tumors are often diagnosed clinically or result in euthanasia without histological confirmation.

In the male reproductive system, testicular neoplasms are common. These are frequently invasive, with seminomas, granular cell tumors, and interstitial cell tumors being the most frequently observed forms [6,16]. In our study, there were two cases of interstitial tumors and one of seminoma. In addition to neoplasms, testicular atrophy is a common biopsy finding in specimens from male rabbits [12]. Our study included one case of testicular atrophy. Reports of primary tumors originating in the gastrointestinal tract or urinary system in rabbits are rare. A case series has reported an instance of rectal prolapse in a pet rabbit caused by leiomyosarcoma [22]. Urothelial carcinoma in the bladder apex was first documented in a pet rabbit in 2018 [23]. In the present study, we identified one case of rectal adenocarcinoma and one case of urothelial carcinoma.

This study has several limitations. First, the sample size was relatively small, which may affect the generalizability of the findings. Future studies involving multiple diagnostic centers would help address this limitation and provide a broader view of tumor prevalence in pet rabbits. Second, only a single molecular method—PCR—was employed for virus detection. Due to the limited amount of available tissue, additional diagnostic approaches such as electron microscopy or immunohistochemistry were not performed. These limitations underscore the need for complementary and more robust detection methods in future studies. Third, a synthetic DNA sequence was used as the positive control for PCR, which does not fully replicate the characteristics of DNA extracted from FFPE tissues. This discrepancy may reduce the assay’s reliability when applied to degraded clinical samples. Fourth, a housekeeping gene was not included in the PCR protocol, so the possibility that DNA degradation contributed to the failure to amplify leporipoxvirus DNA cannot be excluded. Additionally, the present study did not include healthy control tissues, which limits the interpretation of certain findings, particularly in the assessment of virus-associated lesions. This limitation was due to the retrospective nature of the study and the lack of available control samples. Although the neuter status was included in our analysis, other husbandry-related variables were not consistently available and thus were not assessed. These factors may influence tumor development or contribute to regional differences in disease patterns and should be considered in future research. Given these constraints, it is not possible to definitively rule out the presence of leporipoxvirus in Taiwan’s pet rabbit population.

## 5. Conclusions

In conclusion, this study investigated the common diagnoses obtained from surgical biopsies of pet rabbits. The integumentary and reproductive systems were the most commonly affected. We found that males were more likely to develop skin tumors, whereas females were more likely to develop reproductive disorders. No viruses were detected in the cases with fibroma, fibrosarcoma, or myxosarcoma. This suggests that leporipoxvirus does not contribute to the tumorigenesis of these mesenchymal tumors in Taiwan. This study provides an overview of the prevalent conditions in pet rabbits in Taiwan and offers insights that may be applied in the diagnosis and treatment of rabbit malignancies in clinical practice.

## Figures and Tables

**Table 1:** Primer sets used for PCR detection of leporipoxvirus in pet rabbits, adapted from [1].

Primer Sequence (5′→3′)	Expected PCR Product Size (bp)	Annealing Temp. (°C)	No. of Cycles
ACA CCA AAA ACT CAT ATA ACT TCT	220	50	10
CCT ATT TTA CTC CTT AGT AAA TGA T	220	50	20

bp, base pairs; PCR, polymerase chain reaction.

**Table 2:** The signalment of the 70 pet rabbits in Taiwan included in this study.

Age (Months)	n	Sex	n
<6	0	Female, intact	29
6–60	20	Female, neutered	8
>60	50	Male, intact	25
		Male, neutered	6
		Male, unknown neuter status	2

**Table 3:** The 85 diagnoses obtained from the biopsy specimens of 70 pet rabbits classified by the affected system.

Integumentary System			
Neoplastic	n	Non-neoplastic	n
Fibrosarcoma	12	Eosinophilic dermatitis	1
Trichoblastoma	8	Fungal paronychia	1
Fibroma	4		
Lipoma	3		
Mammary gland adenoma	3		
Mammary gland adenocarcinoma	2		
Liposarcoma	1		
Myxosarcoma	1		
Papilloma	1		
Trichilemmoma	1		
Squamous cell carcinoma	1		
Apocrine gland adenocarcinoma	1		
Sweat gland adenoma	1		
Female reproductive system			
Neoplastic	n	Non-neoplastic	n
Uterine adenocarcinoma	16	Endometrial cystic hyperplasia	5
Uterine leiomyoma	2	Uterine adenomyosis	4
Luteoma	2	Cysts of the oviduct	1
Metastatic carcinoma of the oviduct	1	Endometrial venous aneurysm	1
Male reproductive system			
Neoplastic		Non-neoplastic	
Interstitial cell tumor	2	Testicular atrophy	1
Seminoma	1		
Digestive system			
Neoplastic		Non-neoplastic	
Rectal adenocarcinoma	1	Suppurative appendicitis	1
		Suppurative rectitis	1
		Suppurative anusitis	1
		Fatty liver	1
		Hepatic hemorrhage and necrosis	1
		Bile stasis and mineral deposition	1
Urinary system			
Neoplastic			
Urothelial carcinoma	1		

## Data Availability

The data presented in this study are available on request from the corresponding author. The data are not publicly available due to ethical restrictions.

## Supplementary Materials

The following supporting information can be downloaded at https://www.mdpi.com/article/10.3390/ani15091234/s1, Table S1: Case information, including sex, neuter status, tumor location, diagnosis, and PCR testing of pet rabbits.
